# A Time Course Analysis of the Electrophysiological Properties of Neurons Differentiated from Human Induced Pluripotent Stem Cells (iPSCs)

**DOI:** 10.1371/journal.pone.0103418

**Published:** 2014-07-29

**Authors:** Deborah Prè, Michael W. Nestor, Andrew A. Sproul, Samson Jacob, Peter Koppensteiner, Vorapin Chinchalongporn, Matthew Zimmer, Ai Yamamoto, Scott A. Noggle, Ottavio Arancio

**Affiliations:** 1 Department of Pathology & Cell Biology, Columbia University, New York, New York, United States of America; 2 The Taub Institute for Research on Alzheimer’s Disease and the Aging Brain, Columbia University, New York, New York, United States of America; 3 Columbia Stem Cell Initiative, Columbia University, New York, New York, United States of America; 4 The New York Stem Cell Foundation Research Institute, New York, New York, United States of America; 5 Research Center for Neuroscience, Institute of Molecular Biosciences, Mahidol University, Salaya, Nakhonpathom, Thailand; 6 Department of Neurology, Columbia University, New York, New York, United States of America; University of Tampere, Finland

## Abstract

Many protocols have been designed to differentiate human embryonic stem cells (ESCs) and human induced pluripotent stem cells (iPSCs) into neurons. Despite the relevance of electrophysiological properties for proper neuronal function, little is known about the evolution over time of important neuronal electrophysiological parameters in iPSC-derived neurons. Yet, understanding the development of basic electrophysiological characteristics of iPSC-derived neurons is critical for evaluating their usefulness in basic and translational research. Therefore, we analyzed the basic electrophysiological parameters of forebrain neurons differentiated from human iPSCs, from day 31 to day 55 after the initiation of neuronal differentiation. We assayed the developmental progression of various properties, including resting membrane potential, action potential, sodium and potassium channel currents, somatic calcium transients and synaptic activity. During the maturation of iPSC-derived neurons, the resting membrane potential became more negative, the expression of voltage-gated sodium channels increased, the membrane became capable of generating action potentials following adequate depolarization and, at day 48–55, 50% of the cells were capable of firing action potentials in response to a prolonged depolarizing current step, of which 30% produced multiple action potentials. The percentage of cells exhibiting miniature excitatory post-synaptic currents increased over time with a significant increase in their frequency and amplitude. These changes were associated with an increase of Ca^2+^ transient frequency. Co-culturing iPSC-derived neurons with mouse glial cells enhanced the development of electrophysiological parameters as compared to pure iPSC-derived neuronal cultures. This study demonstrates the importance of properly evaluating the electrophysiological status of the newly generated neurons when using stem cell technology, as electrophysiological properties of iPSC-derived neurons mature over time.

## Introduction

Stem cell biology has great potential for the study and treatment of neurodegenerative diseases [1]. The development of technologies to reprogram adult fibroblasts to pluripotent cells, also known as iPSCs [2], [3] has made it possible to generate patient-specific iPSCs. iPSCs derived from patients with neurodegenerative diseases, such as Alzheimer’s [4]–[6], Parkinson’s [7], [8] or Huntington’s [9], [10] disease, are now being used to generate *in vitro* disease models to better understand pathological mechanisms to test potential therapeutics and to investigate the possibility of replacing affected neurons.

There are a variety of methods available to generate neurons through reprogramming of adult cells. For example, upon creation of iPSCs from fibroblasts, neurons can be created in a step-wise fashion, by first transitioning through different intermediate states such as neural progenitors [11], as either embryoid bodies [12]–[15] or adherent cultures [16], [17]. Alternatively, fibroblasts can be transdifferentiated directly to neurons [14], [18]. Neurons generated from these reprogramming protocols clearly express markers reflecting their relative stage of differentiation, such as nestin [19], [20], β-III tubulin [12], [21], MAP2 [22], [23] NeuN [24], synapsin 1 [25] and synaptophysin [24], [26], indicating physiological neuronal development. The expression of the various protein markers used in these studies is not sufficient to fully characterize the developmental progress of neurons. While the use of immunofluorescence has revealed the presence of key neuronal markers, observation of electrophysiological parameters has demonstrated high states of immaturity in iPSC-derived neurons [27]. Electrophysiological properties of neurons are central to their function yet the development of these properties in human iPSC-derived neurons remains largely unknown. Although a few studies have investigated the evolution of the electrophysiological properties of murine iPSC-derived neurons during their maturation from progenitors in mice or rats *in vivo* or *in vitro* [28]–[30], these works have not been fully replicated using human iPSC-derived neurons. Some recent studies have followed the modification of the basal electrophysiological properties of the cells during their differentiation into neurons and their maturation, focusing on particular protocols of differentiation [27] or on the comparison between ESCs and iPSCs [31].

The goal of this study was to characterize the electrophysiological properties in human iPSC-derived forebrain neurons during early time points post-differentiation. We investigated passive and active membrane properties, voltage-gated potassium (K^+^) and sodium (Na^+^) channels, calcium (Ca^2+^) transients, and miniature excitatory post-synaptic currents (mEPSCs) over time, and determined how these measures compare to what has previously been established in dissociated murine cultures [32]–[35]. This fundamental characterization will provide critical information that will not only inform us about the basal electrophysiological properties of human iPSC-derived neurons, but will provide insight into how electrophysiological measurements may be used to evaluate the impact of mutations relevant to neurologic diseases.

## Materials and Methods

### Cell cultures

Cell culture reagents were from Life Technologies (Carlsbad, California, USA) unless otherwise stated. All cell culture media contained penicillin-streptomycin (100 U/mL–0.1 mg/mL). Undifferentiated iPSCs from wild-type (WT) cell line 7889O [15] were kept on irradiated mouse embryonic fibroblasts (MEFs) (Globalstem, Rockville, MD, USA) plated on TC plates with 0.1% gelatin, and grown in human ESC medium (HUESM; 20% KSR/KO-DMEM, 0.1 mM Non-essential amino acids, 2 mM Glutamax, 0.1 mM 2-Mercaptoethanol, 10 ng/ml basic Fibroblast Growth Factor-bFGF). Monolayer neuronal differentiation was carried out in a custom mTesR1 lacking 5 components (TGFβ1, FGF2, GABA, LiCl, and pipecholic acid) found in the standard mTeSR1 compositions (Stem Cell Technologies, Vancouver, BC, Canada, Cat. No. 05892) and Neurobasal media supplemented with B-27 devoid of retinoic acid. SB431542, Y-27632, and LDN-193189 were from ordered from Stemgent (Cambridge, MA, USA).

### Monolayer Neuronal Differentiation

iPSC colonies grown on MEFs were collected under the microscope to remove differentiated cells and pre-plated for 1 h on gelatin coated plates to remove MEFs. The resulting supernatant was centrifuged and subsequently resuspended in mTesR1 media (Stem Cell Technologies) containing 10 µM ROCK inhibitor, Y-27632 (Stemgent). Cells were plated on 6 well plates at a density of 200,000 cells per well, coated with polyornithine (100 µg/mL) and laminin (3 µg/mL) (POL) and allowed to recover for 3 days to achieve confluency. iPSCs were differentiated into neurons with dual-smad inhibition between day 0 and day 9 in custom mTesR1 using 10 µM SB431542 and 250 nM LDN193189, where day 0 corresponds to the start of dual-smad inhibition. Inhibition of both branches of TGFβ signaling is sufficient to promote neuronal fate, and saturates by 10 days of inhibition [36]. At day 9, cells were detached with acutase, split and plated onto POL coated dishes with the same conditions as the initial plating. Medium was changed from custom mTesR1 to Neurobasal + B27 supplement ([-] retinoic acid) in a stepwise fashion, and cells were fed every 2–3 days. The same procedure was employed at day 25–28, when cells were re-plated onto POL coated 18 mm glass coverslips (Waldemar Knittel Glasbearbeitungs, Germany) contained in 12 well plates at a density of 100,000 cells/well. Cells were treated as before with the addition of BDNF (40 ng/mL; R&D Systems, Minneapolis, MN, USA) to the culture medium. All cells utilized in these studies including electrophysiological, imaging, FACS and immunocytochemical experiments were prepared using this protocol.

### Co-cultures with mouse neonatal glia

Neonatal glial cultures (a mixed population of microglia, astrocytes and oligodendrocytes) were prepared from 2-day-old C57Bl6J mouse pup cortices. Following decapitation, the skulls were removed using sterile dissecting scissors and the brain was placed in ice-cold MEM in 100 mm Petri dish. Olfactory bulbs and cerebellum were removed. Cortices were separated from the rest of the brain, and meninges were carefully removed. Cells were dissociated through enzymatic treatment (0.25% trypsin) with the addition of 10 µM DNase and subsequent trituration. Cortical cells were grown in T-75 flasks in a medium containing 90% DMEM, supplemented with 10% fetal bovine serum at a concentration of 10,000 viable cells/mL with no coating. Under these conditions, primary neurons did not attach to the plastic surface. To remove all neurons, cells were passaged three times, once every week, until confluence was achieved. Medium was exchanged twice a week. One day prior to the second split of the iPSC-derived cultures, glial cultures were treated with 10 µM arabinofuranosylcytosine (AraC; Sigma, St. Louis, Missouri, USA) to inhibit further growth. At the time of the second split (day 25–28), the iPSC-derived cells were detached as explained in the “Monolayer Neuronal Differentiation” section, mixed with the mouse glia (glia:neuron ratio = 2∶1) and immediately re-plated on uncoated 18 mm glass coverslips contained in 12-well plates at a concentration of ∼45,000 cells/cm^2^ (15,000 neurons and 30,000 glial cells). After 24 h, culture medium was exchanged, and subsequently half of it was replaced every 3 days.

### Ethics statement

The following protocol was approved by the Institutional Animal Care and Use Committee (IACUC) of the Columbia University (Protocol Number: AC-AAAB6858). iPSCs were established from previously published de-identified biobanked fibroblast lines as previously described [37].

### Experimental Design

**Figure 1:**
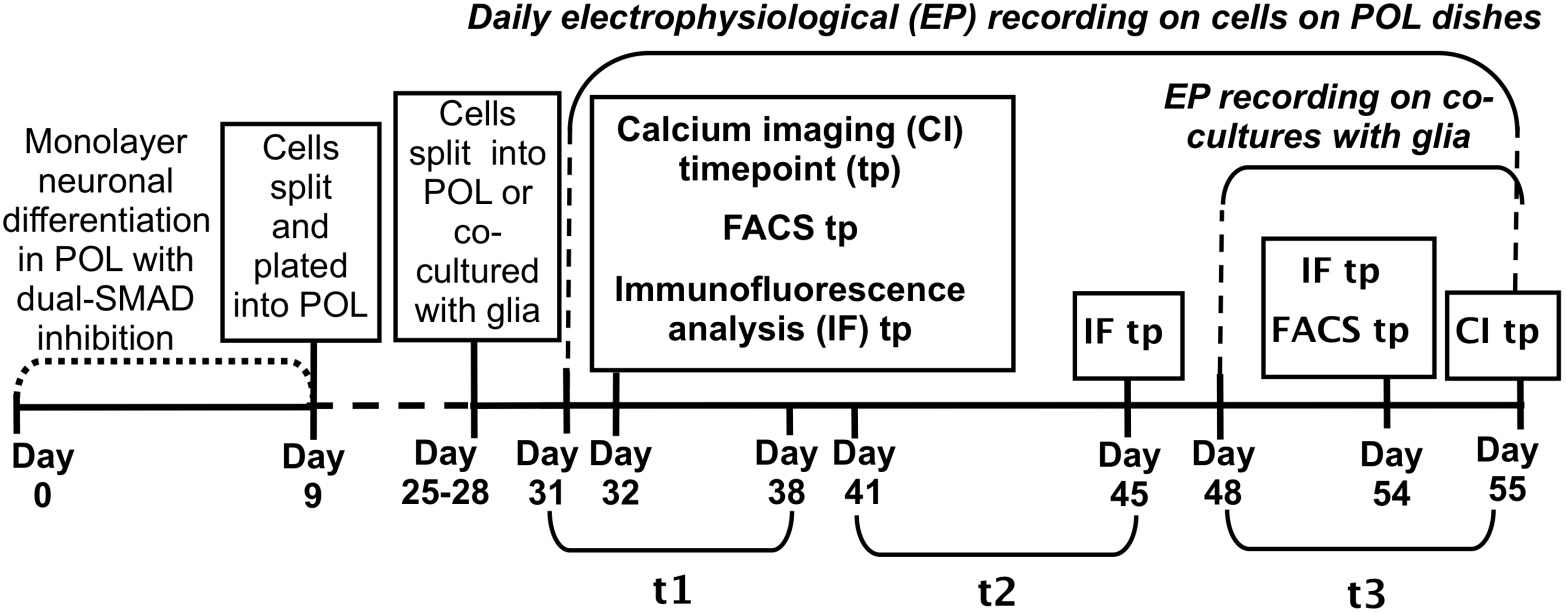
Timeline of the experiments. The different steps of the experiments are represented, as well as the time-points for the electrophysiological, calcium imaging, immunofluorescence and FACS analyses. The 3 different timing groups for statistical purposes are the following: t1  =  from day 31 to day 38; t2  =  from day 41 to day 45; t3  =  from day 48 to day 55.

After the differentiation process we analyzed the cells from day 31 to day 55 through immunocytochemistry, patch clamp technique, and calcium imaging experiments. We chose this range because our initial attempts to record APs from earlier time points (<day 31) revealed that these neurons do not fire APs. For the comparison between the cultures on POL surface and the co-culture with mouse glial cells, we analyzed their electrophysiological parameters from day 48 to day 55. A timeline of the experiments is shown in Figure 1.

### Immunocytochemistry

Immunostaining was performed as described before [38]. Hoechst 33342 (Sigma) was used to visualize DNA. Antibodies against the following proteins were used: microtubule-associated protein 2 (MAP2) (1∶1000, Abcam, Cambridge, MA, USA), neuron-specific class III beta-tubulin (Tuj1) (1∶500, Covance, Princeton, NJ, USA), postsynaptic density protein 95 (PSD95) (1∶200, Invitrogen), forkhead box protein G1 (FOXG1) (BF1, 1∶300, Abcam), FORSE-1 (1∶100, Developmental Studies Hybridoma Bank), T-box, brain 1 (Tbr1) (1∶200, EMD Millipore, Darmstadt, Germany), and Oct4 (1∶500, Stemgent). The percentage of BF1 + cells at day 32 of differentiation was scored by analyzing three random fields per well and averaging the percentage of BF1 + cells, for three biological replicates (SEM reflects biological replicates). An average of 229 cells were scored per field.

### Flow Cytometry

Cells from day 32 of neuronal differentiation were subjected to flow cytometry analysis as has been done previously [37], for CD56 and CD24 (1 µL each, BD Biosciences, San Jose, CA, USA). SEM reflects three biological replicates.

### Patch clamp analysis

The whole-cell ruptured patch clamp technique was used to record from 268 neurons from day 31 to day 55. Neurons co-cultured with neonatal mouse glial cells were recorded from day 48 to day 55 (t3). Recordings were performed at room temperature under continuous perfusion (1 ml min^−1^) with a bath solution containing (in mM): NaCl (119), KCl (5), HEPES (20), glucose (30), MgCl_2_ (2), CaCl_2_ (2) and glycine (0.001). The osmolarity of the solution was adjusted to 310 mOsm with sucrose, and the pH was adjusted to 7.3. Single cells were selected for recordings based on a spherical and bright cell body and the presence of 2 or more neurites, viewed through a phase contrast objective on a TS100 Eclipse microscope (Nikon, Tokyo, Japan). In some experiments, 1 µM tetrodotoxin (TTX), 10 mM tetraethylammonium (TEA) or 0.1 mM picrotoxin were added to the bath solution to block Na^+^ channels, K^+^ channels or γ-aminobutyric acid (GABA) receptors, respectively, through the perfusion system. Glass electrodes (World Precision Instruments, Sarasota, FL, USA) were pulled with a PIP5 pipette puller (Heka Elektronik, Lambrecht/Pfalz, Germany) (resistance between 5.5–7 MΩ), and filled with an intracellular solution containing (in mM): K-Gluconate (130), KCl (10), HEPES (5), CaCl_2_ (0.06), MgCl_2_ (5), EGTA (0.6), ATP (2), GTP (0.2), phosphocreatine (20) and leupeptine (0.2) and 50 U/ml creatine-phosphokinase. Recordings were made with a PC-501A amplifier (Warner Instruments, Hamden, CT, USA). Signals were filtered at 1 kHz, sampled at 10 kHz with 16-bit resolution with a Digidata 1440A (Molecular Devices, Sunnyvale, CA, USA). Access resistance was constantly monitored throughout all experiments. In all recorded cells, RMP was assessed immediately after the achievement of the whole-cell configuration. The calculated liquid junction potential (LJP) with our solutions was −5.8 mV and all data were corrected accordingly.

We used three recording protocols:

1. Voltage-clamp steps (from −90 mV to +20 mV in 10 mV increments and 1-s in duration) from a starting potential of −70 mV to investigate the presence and amplitude of Na^+^ and K^+^ currents in the presence and/or absence of 1 µM TTX and/or 10 mM TEA to block Na^+^ and K^+^ currents, respectively;
2. Current clamp steps (0 pA for 100 ms, steps from −60 pA to +120 pA, 20 pA each, for 1 s) to verify the ability of the cells to generate APs in response to depolarization current steps and the voltage-gated Na^+^ channel-dependence of the APs in the presence of 1 µM TTX;
3. Voltage clamp at −70 mV for 2 min up to 10 min with 1 µM TTX to record spontaneous post-synaptic currents.

Data analysis was performed using Clampfit 10 software (Molecular Devices) and Matlab 8.0 (MathWorks, Natick, MA, USA). The following electrophysiological parameters were examined: resting membrane potential (RMP), percentage of cells with APs in current-clamp mode over the total amount of cells recorded, percentage of cells with mEPSCs over the total amount of cells recorded in voltage-clamp mode, amplitude of the negative peak of inward Na^+^ currents, amplitude of the peak of outward K^+^ currents, full-width at half maximum (FWHM) of APs (calculated as the average of the duration of the APs at half-maximal spike amplitude), AP threshold (determined as the 1st peaks in the 2nd derivative of the voltage function in response to the minimum current step able to elicit an AP) and AP amplitude (calculated in current-clamp mode for the first elicited AP). In addition, the membrane time constant (τ) and the input resistance (Rin) were estimated in current-clamp mode through the voltage responses of the cells to injected hyperpolarizing current steps of −20 pA. Rin was derived from the linear portion of the current-voltage plot. τ was calculated by minimizing the squared deviation between the function f (t)  =  V_0_− (V_0_ · e^(t/τ)^) and the data, where V_0_ is the steady state response. mEPSCs were analyzed with the MiniAnalysis program designed by Synaptosoft Inc. (Decatur, GA, USA). Amplitude threshold for event detection was 4 pA. Each event was visually inspected and only events with a distinctly fast-rising phase and a slow-decaying phase were accepted. Rise and decay times were defined as the interval between the very beginning of the rising phase and the peak (rise time), and the interval between the peak and 90% of the decaying phase (decay time).

### Calcium Studies

Neuronal cultures from iPSC lines that had been neuronally differentiated for 32 or 55 days were pretreated with an HBSS loading buffer containing 20 mM HEPES, 2.5 mM probenecid, and Fluo-4NW (Life Technologies) for 30 min at 37°C and then at room temperature for an additional 10 min before imaging, following the manufacturer’s instructions. Cells imaged were plated and treated under the same conditions as cells used for electrophysiological experiments.

Cells were then placed at room temperature in an imaging chamber (Warner Instruments Recording/Perfusion Chamber, RC-22) and perfused at a rate of 1–2 ml min^−1^ with the same bath solution used for the electrophysiological analysis. Where needed, the solution in perfusion was changed with solutions containing 1 µM TTX to block Na^+^ channels. Within a given ROI (Region Of Interest), only active cells were chosen for analysis. Cells were imaged on a Nikon BX51W1 with a 20X objective (0.5 n.a.) and a Hamamatsu Orca R2 camera (Hamamatsu, Hamamatsu City, Japan). Data was captured and analyzed with HCI Image (Hamamatsu) using the DIA plug-in. Time-lapse images were taken every 1.5 seconds for 5 minutes using 512×512 pixel resolution and cells were excited using the 488 nm laser line. ROIs (4 µm×4 µm) were drawn over each cell soma and change in fluorescence intensity over time was calculated within the imaging software. All signals were background subtracted. Ca^2+^ transients were analyzed manually and with a custom written routine in Matlab (Mathworks), based on the “peakfinder” and “relevantpeaks” functions.

### Electrophysiology statistical analysis

Data are expressed as mean ± SEM (calculated as the rate between standard deviation and the square root of the sample size - assuming statistical independence of the values in the sample).

To facilitate statistical analysis data were clustered in the following 3 groups: from day 31 to day 38 (t1); from day 41 to day 45 (t2); from day 48 to day 55 (t3).

Each data set was tested for normality with the Anderson-Darling normality test. If the normality of the data was confirmed with a 90% confidence interval, comparison of electrophysiological parameters at different time points was performed using one-way analysis of variance (ANOVA) and the Student’s t test with a level of significance set for p<0.05 was used to compare results on synaptic transmission of neurons grown on plastic with those grown in co-cultures with glia. Where the ANOVA output was significant (p<0.05), the Tukey post-hoc test was used to provide specific information on which means are significantly different from each other. If the samples tested had an Anderson-Darling p value higher than 0.1 (data not normal), the non-parametric ANOVA Kruskal-Wallis test was run to compare the parameters at different time points, and the Wilcoxon rank sum test was used to compare results between neurons grown on plastic with those grown in co-culture with glia. Where significance was achieved with the non-parametric ANOVA Kruskal-Wallis test, the non-parametric Nemenyi Test was used as post-hoc analysis to determine which means are significantly different from each other.

## Results

### Immunofluorescent examination of iPSC-derived neurons

**Figure 2:**
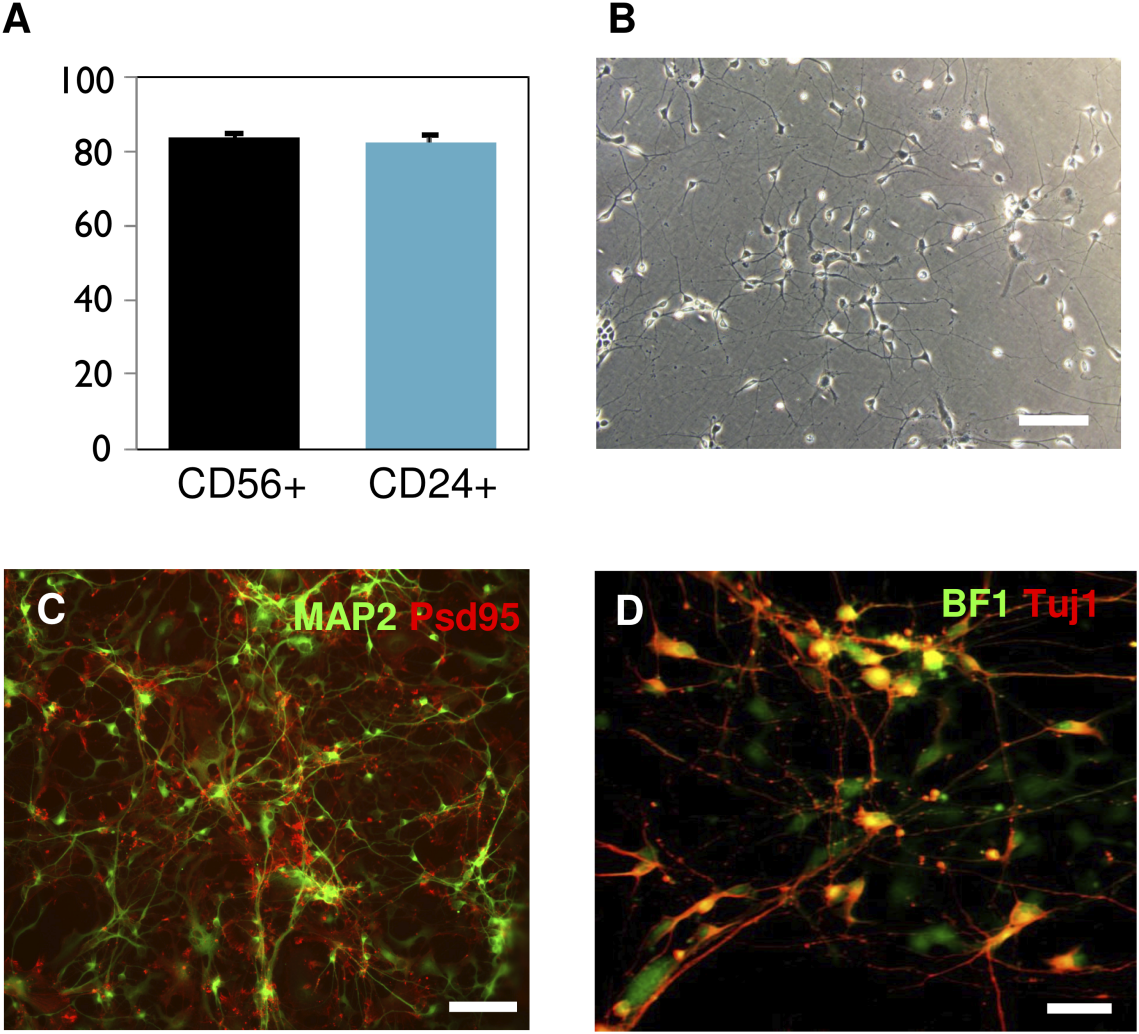
Flow cytometry and immunofluorescence analyses of iPSC-derived neurons. A) Flow cytometry analysis of neuronal surface markers CD56 and CD24 at day 32 of differentiation. B) Representative phase contrast microscope image of cultured neurons at day 32 of differentiation. Scale bar: 100 µm. C) At day 45 of differentiation, cells express MAP2 (green) along soma and dendrites and the postsynaptic protein PSD95 (red), confirming the advanced stage of differentiation into neurons. Scale bar: 100 µm. D) Cells also express the forebrain-specific marker BF1 (green) and Tuj1 (red) at 45 days of culture. They are often co-localized (yellow). Scale bar: 50 µm.

Neurons were generated from iPSCs using a standard method (with slight modification) that relies on the inhibition of both branches of TGFβ signaling in undifferentiated pluripotent cells [17], [37]. A timeline describing our neuronal differentiation and analysis can be found in Figure 1. At the start of our time course (day 32), approximately 80% of the cells were neuronal as measured by flow cytometry analysis of CD56+ (84.1%±0.9%) and CD24+ (82.4%±1.9%) neuronal surface markers (Figure 2A). Some late stage progenitors do express CD56 [38]. At this time point and throughout the study nestin+neural progenitors were present (Figure S1). Neurons for subsequent electrophysiological analysis were easily distinguishable by phase contrast microscope (Figure 2B). Differentiating cells expressed typical neuronal markers including MAP2 and TuJ1 (Figure 2C–D), further indicating that they are committed to a neuronal lineage. PSD95, a marker of late stage differentiation, was also expressed (Figure 2C). Moreover, a high percentage of neurons expressed the adult forebrain neuron-specific protein BF1 (83%±1.44%), also called FOXG1, which was mainly localized in the nucleus/soma (Figure 2D). Cells were also positive for additional forebrain markers including FORSE-1 and Tbr-1 (Figure S1). Cells were negative for endoderm (AFP) and mesoderm (MF20) germ layer markers, as well as the pluripotency marker Tra160 (data not shown). A few rare cells were positive for Oct4 (Figure S1), perhaps indicating transgene reactivation in a small minority of cells.

### Assessment of neuronal maturation through electrophysiological properties

**Figure 3:**
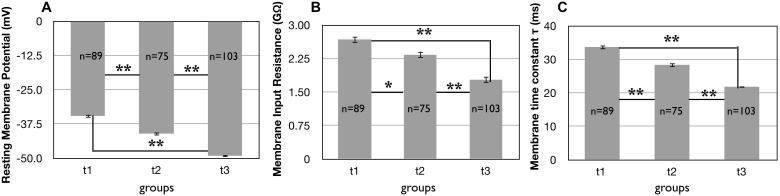
Evolution of basal membrane properties over time in iPSC-derived neurons plated on POL. A) The average resting membrane potential (RMP in mV) decreased over time. The number of cells examined for these experiments are shown inside the bar in these and the following figures. The vertical lines correspond to the SEM in this and the following figures. **: p<0.001; *: 0.001<p<0.05; NS: p>0.05 for these and the following figures. B) The membrane input resistance decreased over time (GΩ). C) The membrane time constant decreased over time (ms).

Since the presence of neuronal markers indicated that our differentiation protocol was successful, we next sought to characterize the basal physiology of the neurons. As a neuron differentiates and matures, the expression of ion channels, such as voltage-gated K^+^ channels (K_v_) and Na^+^ channels (Na_v_), shifts the RMP towards more negative potentials [39], [40], until it reaches about −65 mV in a mature neuron [41]. At this stage, the expression of specific Na_v_ and K_v_ in significant amounts drives the ability of neurons to generate APs. In our iPSC-derived neurons the RMP became more negative over time (Figure 3A). At t3 neurons showed a RMP equal to 49.08±0.5 mV (n = 103), and the post-hoc analysis revealed that the value was significantly more negative than the RMP at t1, and t2 (Figure 3A).

As neurons mature, two passive electrical membrane properties, the input resistance R_in_ and the membrane time constant τ, decrease and shorten, respectively [42], [43]. Consistent with these observations, cells initially showed a relatively high R_in_ (Figure 3B) and long τ values (Figure 3C) both of which changed value consistent with neuronal maturation over time. Post-hoc analysis revealed significant differences between all groups for R_in_ and τ (Figure 3B–C, respectively).

**Figure 4:**
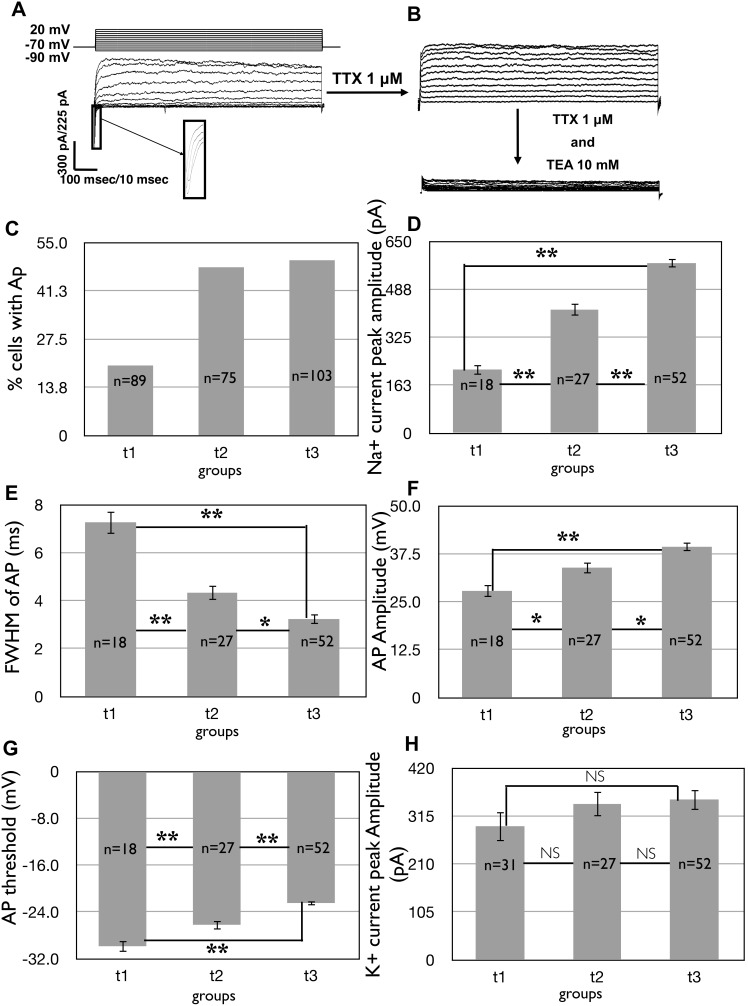
Evolution of active membrane properties over time in iPSC-derived neurons plated on POL. A) Representative traces of voltage clamp recordings showing fast inward currents followed by long-lasting outward currents in a cell at day 55, due to voltage steps in 10 mV increments (shown in the upper panel). The inset shows a high magnification view of the inward current. Inward Na^+^ currents were observed in 13 out of the 22 cells. Following initial recording, cells were perfused with 1 µM TTX to block Na^+^ currents, and subsequently with 10 mM TEA (tetraethylammonium) to block K^+^ currents. B) The percentage of cells showing APs in response to step current injections of 20 pA over the total number of cells recorded in the same day. C) The graph shows the increase over time of the average amplitude of the inward Na^+^ current measured at the peak (pA). D) The graph shows a decrease over time of the Full Width at Half Maximum (FWHM) (ms) of APs. E) The graph shows an increase of the AP amplitude (mV) over time. F) Evolution of the AP threshold (mV) over time. G) Evolution of the average peak K^+^ currents over time (pA).

During neuronal maturation, the expression of Na_v_ increases and the membrane becomes capable of generating APs following depolarization [27], [44], [45]. We observed a small percentage of cells (20%) capable of producing APs (and inward sodium currents) upon depolarization at t1. The percentage of cells capable of producing APs increased with time, reaching 50% at t3 (Figure 4B). The increase in the number of Na_v_ over time was also confirmed by the value of the average peak Na^+^ current (Figure 4C). This value increased 2.67 folds between t1 and t3 and the increase was statistically significant for each group with respect to the previous ones (Figure 3C).

Measurement of the FWHM of APs also showed a decrease over time. At t1 the average value of FWHM was 7.27±0.49 ms (n = 18), whereas at t3 it was 3.23±0.22 ms (n = 52) (Figure 4D). Consistent with these findings, AP amplitude and threshold, two other parameters connected to the active membrane properties, changed over time (AP amplitude: 27.83±1.65 mV, n = 18 at t1 and 39.33±1.2 mV, n = 52 at3; AP threshold: −29.86±1.01 mV, n = 18 at t1 and −22.48±0.44 mV, n = 52 at t3, Figure 4E–F).

In order to confirm the involvement of Na_v_ in the observed currents, we applied the selective Na_v_-blocker TTX. No inward currents were recorded after its application (Figure 4A), confirming that Na_v_ were responsible for the inward currents. Moreover, the combined treatment of 1 µM TTX and 10 mM TEA to block also K_v_, suppressed all currents (Figure 4A) demonstrating that the outward currents we observed in iPSC-derived neurons were mainly due to K^+^ ion flux through the cell membrane. We did not observe a monotone increase or decrease in the amplitude of the K^+^ current during the development of iPSC-derived neurons using our protocol (Figure 4G). As mentioned in the methods section, no K^+^ currents were observed in the cells that we recorded between day 15 and day 20 (data not shown). This suggests that these currents and subsequent large-scale expression of K^+^ channels sufficient for AP formation are not likely to appear until at least after day 20. After day 20, K^+^ currents do not significantly reflect changes in neuronal maturation until late time points (> day 55).

**Figure 5:**
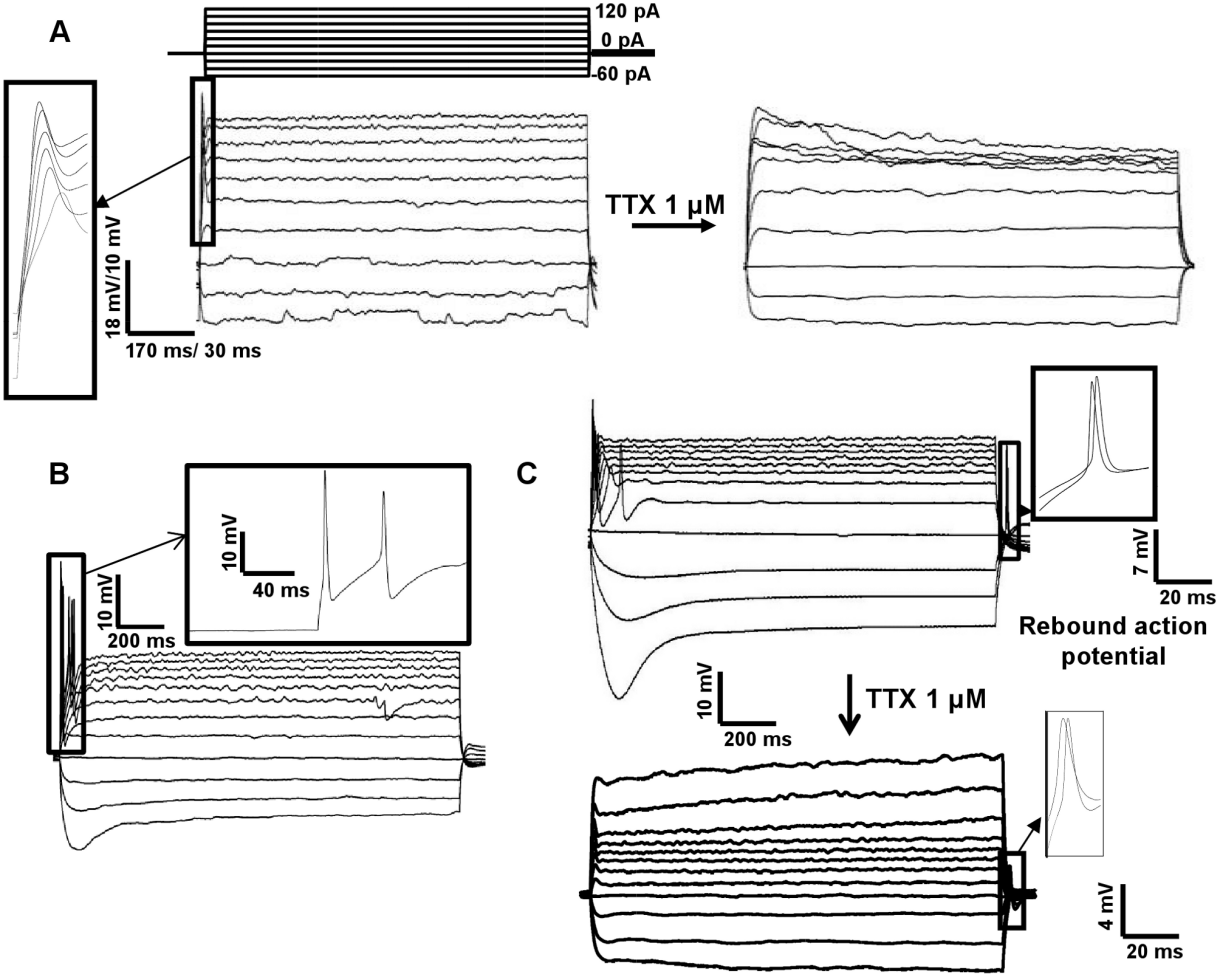
Characterization of the APs in iPSC-derived neurons plated on POL. A) Representative APs in response to step current injections of 20 pA (upper panel) in current clamp mode. The cell shown was recorded at day 45. APs were observed in 4 out of 10 cells. Following initial recording, APs were blocked by 1 µM TTX. B) Multiple firing of APs in response to step current injections in 20 pA increments was observed in cells from day 45. The trace shown in this figure was obtained from a cell recorded at 55 days. The inset shows a high magnification view of the APs. C) At day 55, 13 out of 22 cells had APs, and 3 of them had rebound APs like the one shown on the inset of this panel. Multiple APs in response to depolarizing current injections and “rebound” APs at the end of hyperpolarizing current injections were also visible. After perfusion with 1 µM TTX, the APs, due to Na^+^ channel dependence, was blocked, but rebound APs persisted in 4 out of 17 cells with rebound APs.

Step current injections of 20 pA in current-clamp mode elicited only single APs at t1 and t2 which were blocked by 1 µM TTX (an example in Figure 5A at day 45). Consistent with previous studies [27], cells in this phase of maturation were not able to produce trains of APs. At t3, 50% of cells had APs that were blocked by perfusion with 1 µM TTX indicating that they were due to the opening of Na^+^ channels, and 30% of them produced multiple APs in response to a prolonged depolarizing current step (an example in Figure 5B at day 55).

In addition, we observed single or multiple rebound APs in the course of neuronal differentiation. These APs occurred upon termination of hyperpolarizing pulses in current-clamp mode (Figure 5C). At t3 27% of cells firing had a rebound AP (an example in Figure 5C, inset, at day 55). In addition, rebound APs were TTX-resistant in 23% of neurons, suggesting the involvement of Ca^2+^ channels which are also known to produce inward currents, and/or inward rectifier currents in the generation of rebound APs [46].

### Assessment of spontaneous calcium transients in iPSC-derived neurons

**Figure 6:**
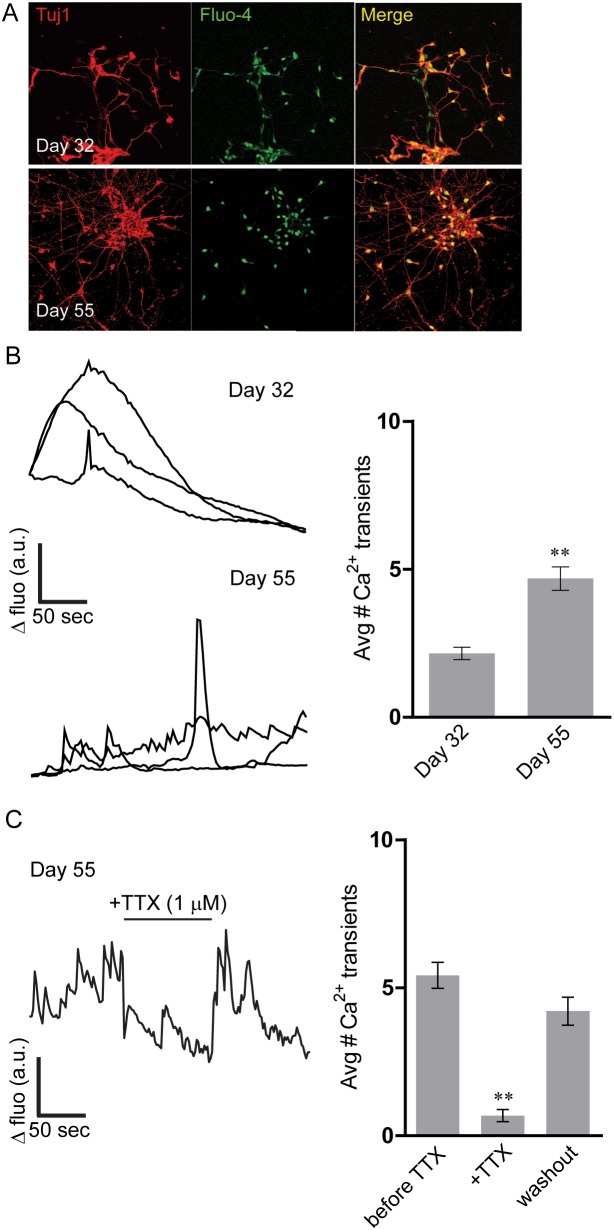
Spontaneous calcium transients increase in iPSC-derived neurons during development. A) After imaging of spontaneous Ca^2+^- transients iPSC-derived neurons were fixed and stained for Tuj1 at day 32 and day 55, revealing more complex morphology at day 55 (scale bar = 40 µm). B) Representative Fluo-4 spontaneous Ca^2+^- transients from 3 iPSC-derived neurons at day 32 and at day 55 (ΔFluo = ΔFluorescence; a.u. = arbitrary unit). Neurons exhibit slow broad transients, consistent with early developing neurons at day 32. By day 55, calcium transients take on a more spike-like morphology reflecting what has been reported for maturing neurons. Group data demonstrates that iPSC-derived neurons exhibit a significant increase in calcium transients at day 55 as compared to day 32 (n = 113 neurons/10 coverslips for both groups, p<0.001 comparing day 32 with day 55). C) The average number of Ca^2+^ transients at day 55 was dramatically reduced following perfusion with 1 µM TTX with recovery following washout of the toxin (n = 20/3 coverslips, p<0.001 compared with average number of Ca^2+^ transients prior to TTX perfusion). A sample trace derived from a representative experiment is shown on the left).

Large-scale synchronous network activity, comprised of barrages of AP-induced network-wide Ca^2+^ transients, is an integral part of the development and functional maintenance of brain networks and mature synapses [47]. The balance of neuronal excitation and inhibition is in part driven by Ca^2+^ transients, which regulate activity-dependent gene expression and control neurotransmitter release via AP width. Developmentally, it has been demonstrated that Ca^2+^ transients play an important role in driving differentiation [48] and that the timing and frequency of Ca^2+^ transients reflect stages of neuronal maturation [47]. We next asked whether our neurons displayed Ca^2+^ transients and whether the frequency of these transients changed during the course of iPSC-derived neuronal development. To measure cytosolic Ca^2+^ we bath-applied Fluo-4NW to iPSC-derived neurons at day 32 and also at day 55, and recorded spontaneous Ca^2+^ spikes during a 5 min interval (Figure 6). Morphologically, neurons became more complex over time and this morphological complexity coincided with more frequent Ca^2+^ signals (Figure 6A). At 32 days after initial plating and differentiation, we observed a low average number of transients (2.2 transients/5 minute interval; n = 113 neurons/10 coverslips, Figure 6B). The Ca^2+^ transients we observed at this early time point were morphologically broad. However, similar to the electrophysiological parameters investigated above, Ca^2+^ transients matured over time, such that when we studied iPSCs at day 55, Ca^2+^ transients displayed more spike-like morphology and the average number of events increased significantly (4.7/5 minute interval; n = 113 neurons/10 coverslips, Figure 6B). These changes were accompanied by an increase in the percentage of active cells (31.9% of the total at day 32 vs. 56.5% of the total at day 55). Finally, in additional experiments, as previously demonstrated [37], perfusion of the cultures with 1 µM TTX abolished most of the Ca^2+^ transients at day 55. These recovered after subsequent washout (n = 20 neurons/3 coverslips, Figure 6C) suggesting that they were dependent upon activity. Moreover, the presence of transients recovered after TTX washout shows that inhibition of activity is not a coincidental artefact of phototoxicity. The small number of TTX-resistant transients that remained during application of TTX could be either due to spontaneous release of neurotransmitters, or to TTX-resistant action potentials.

Taken together, these data are consistent with the development of the electrophysiological parameters shown above, including RMP, and AP properties. This suggests that as firing activity increases and RMP decreases during development, Ca^2+^ transient frequency increases and their duration shortens. Furthermore, these data agree with observations made in both cultured cortical neurons and neurons derived from human iPSCs [49], [50].

### Assessment of miniature excitatory post-synaptic currents (mEPSCs) in iPSC-derived neurons

**Figure 7:**
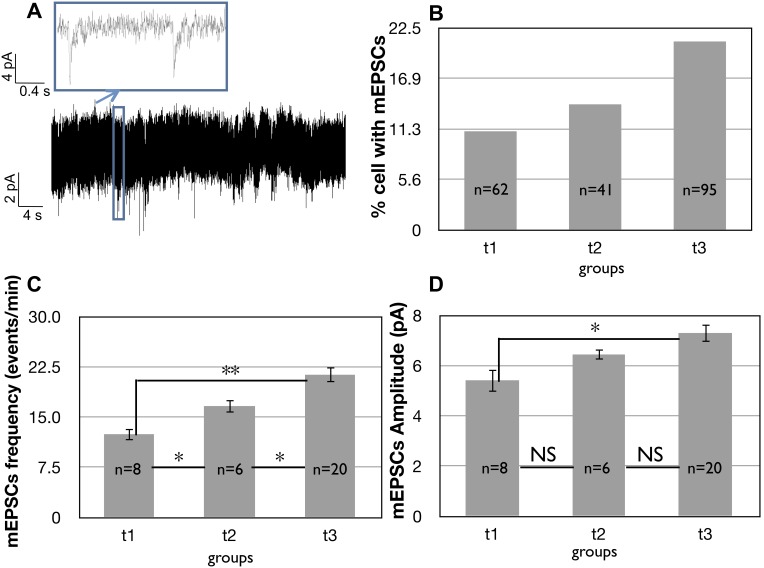
Evolution of synaptic activity due to AP-independent release of neurotransmitters over time in iPSC-derived neurons plated on POL. A) Example of continuous recording from an iPSC-derived neuron at 55 days (holding potential at −70 mV). The inset shows an enlarged view of two spontaneous post-synaptic events. B) The number of cells showing synaptic events over the total number of cells recorded in the same day. Cells were recorded at −70 mV in 1 µM TTX in voltage clamp. C) Increase over time of the frequency of the mEPSCs, measured as number of spontaneous events/minute. D) Evolution of the mEPSC amplitude with a voltage clamp at −70 mV from t1 to t3.

To investigate synaptic development of iPSC-derived neurons, we determined the percentage of cells with mEPSCs over the total amount of cells recorded at every time-point in the presence of TTX to block APs (Figure 7A). Approximately 11% of neurons displayed mEPSCs at t1. Interestingly, at early developmental stages, mEPSCs were not blocked by the application of (2R)-amino-5-phosphonovaleric acid (D-APV) (50 µM) and 6-cyano-7-nitroquinoxaline-2,3-dione (CNQX) (20 µM), two antagonists for N-methyl-D-aspartate (NMDA) and 2-amino-3-(5-methyl-3-oxo-1,2-oxazol-4-yl) propanoic acid (AMPA)/kainate receptors, respectively. Additionally, no cells responded to pressure application of 1 µM glutamate (data not shown). By contrast, mEPSCs were blocked by the reversible GABA_A_ antagonist bicuculline (10 µM), suggesting that during early maturation of iPSC-derived neurons mEPSCs may rely on GABAergic neurotransmission.

The percentage of cells exhibiting mEPSCs increased over time (Figure 7B). At t3, we found mEPSCs in 21% of neurons, and 40% of the cells exhibiting mEPSCs were blocked by the NMDA receptor antagonist D-APV (50 µM). Bicuculline application had no effect on mEPSCs at that time point, suggesting that as neurons derived using our protocol mature, mEPSCs are no longer dependent upon GABA_A_ receptors.

We also measured the developmental changes in frequency and amplitude of mEPSCs over time. At t1 the average frequency was 12.45±0.95 events/min (i.e. 0.2 Hz, n = 8, Figure 7C). This value increased over time, reaching an average of 21.32±1.15 events/min (i.e. 0.36 Hz, n = 20) at t3. Using post-hoc analysis we found that the increase in the frequency of mEPSCs was statistically significant for each group with respect to the previous ones (Figure 7C). In addition to the frequency of mEPSCs, the amplitude also increased over time from 5.42±0.47 pA (n = 8) at t1 to 7.3±0.37 pA at t3 (n = 20) (Figure 7D). Post-hoc analysis revealed a significant increase in the amplitude of events from t1 to t3 (Figure 7D).

### Co-culturing with glial cells enhances neuronal development

**Figure 8:**
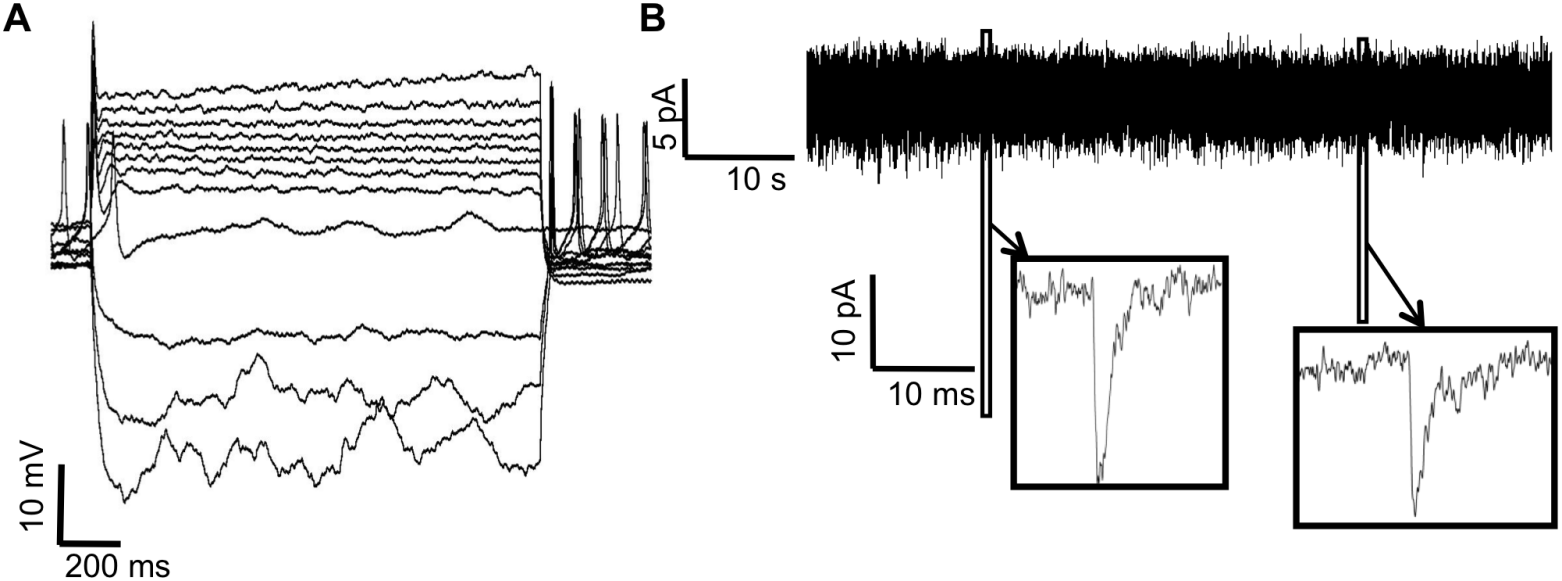
iPSCs-derived neurons plated with glia express different electrophysiological properties than neurons plated on POL. A) Representative APs in response to step current injections of 20 pA in current clamp mode for a cell at day 55 co-cultured with mouse glia. APs were observed on 13 out of 15 cells, and in 4 out of the 13 cells with APs rebound APs at the end of hyperpolarizing current injections were also present. B) Examples of mEPSCs recorded in voltage clamp configuration. Cells were plated on mouse glia by mixing them before plating. They were held at −70 mV. TTX at a concentration of 1 µM was added to the bath solution to suppress spontaneous excitation and to allow isolation of synaptic events induced by spontaneous transmitter release.

The presence of glial cells is known to facilitate differentiation and maturation of cells into neurons [51], regulating neuronal synaptic development and connectivity [52] as well as synaptic activity through the release of glutamate or ATP [53], [54] and cell contact molecules such as ephrins [55], [56]. A recent study reported that astroglial substrate greatly accelerates neurodevelopmental progression of human iPSC-derived neurons [57]. We therefore co-cultured iPSC-derived neurons with mouse glial cells. Analysis of electrophysiological properties of the cells co-cultured with neonatal glial cells at 55 days revealed an acceleration of the development of various electrophysiological parameters including RMP, AP capability and Na^+^ current amplitude (n = 15 co-cultured cells vs. 20 POL-plated neurons; Figure 8A and Table 1). The average value of RMP was −60.55 mV in co-cultured cells compared to −51.7 mV in POL-plated neurons. Additionally, almost 90% of the neurons were able to produce APs in response to depolarizing currents compared to 60% for POL-plated neurons. The average peak of inward Na^+^ currents in the co-culture model was higher, although this difference did not reach statistical significance (488±116.2 pA, n = 15 for POL-plated neurons vs. 604±116.3 pA, n = 20 for co-cultures, Table 1).

**Table 1:** Electrophysiological parameters in neurons plated on POL and mouse glia co-cultures at day 55.

	POL 55 days		Co-culture		P value
	Mean	SE	Mean	SE	
Resting membrane potential (mV)	−51.7	2.51	−60.55	5.61	<0.01
Percentage of cells with APs	60%	n/a	86%	n/a	n/a
Na^+^ current peak amplitude	604	116.3	488.66	116.2	>0.05
mEPSC frequency (number events/min)	23.85	4.43	67.1	24.45	0.013
mEPSC amplitude (pA)	8.95	0.73	11.15	1.85	>0.05
mEPSC rise time (ms)	1.001	0.03	0.974	0.09	>0.05
mEPSC decay time (ms)	3.31	0.17	3.645	0.46	>0.05

The table summarizes the differences between various electrophysiological properties in neurons plated on POL (n = 20) and mouse-glia co-cultures (n = 15) at day 55. Values are expressed as mean and SE (standard error).

Next, we analyzed synaptic properties of neurons co-cultured with glia in the presence of TTX. We found mEPSCs in 26.6% of iPSCs co-cultured with glial cells vs. 20% for POL-plated neurons (4 of 15 for cells co-cultured with glia and 4 of 20 for cells plated on POL; Table 1). Interestingly, these synaptic events were not blocked by the GABA_A_ antagonist bicuculline (10 µM), whereas they were blocked by the NMDA receptor antagonist D-APV (50 µM) in 2 cells, suggesting that they were due to excitatory transmission. Most importantly, the mEPSC frequency was around 3 fold higher than in POL-plated neurons (Table 1) with an average amplitude equal to 11.15±1.85 pA in cells on glia compared to 8.95±0.73 pA in cells plated on POL (Table 1). No statistically significant differences were detected in rise time and decay time between the cells plated on POL and the ones with glia mix at day 55 (Table 1).

## Discussion

The establishment of reliable *in vitro* systems for the modelling of neurodegenerative disorders has been a major challenge for studying pathologic mechanisms, screening new drugs, and developing new therapies using human stem cells. Similar to human ESCs, human iPSCs derived from somatic cells possess self-renewal and pluripotency properties and are expected to serve as a powerful tool to model diseases for basic and translational research [58]–[62]. If neurons derived from iPSCs are to be useful for modelling human neuron development and function, it is important that they acquire mature functional characteristics similar to neurons *in vivo*. Here we analysed the differentiation of iPSCs and their maturation into neurons following a time dependent course of their electrophysiological properties. We found that cellular maturation culminated in functional physiological properties typical of mature neurons, both for passive and active membrane properties: the RMP decreased over time, as well as the R_in_ and τ. The number of cells responding to a depolarizing voltage step with opening of Na^+^ channels and exhibiting an inward current increased over time. We compared the RMP observed in our culture system with other values calculated for neurons derived from mouse embryonic cells [63]. At early time points (t1) we found that the RMP of iPSC-derived neurons was more negative (−34.56 mV) than that recorded in mouse embryonic cells (−23 mV) [63]. Aside from possible differences due to differentiation protocols or starting organism, the difference between our findings can partially be explained by the use of a five-fold greater intracellular Cl^−^ concentration in the patch solution used by Sonoda et al. [63]. On the contrary, at later stages (t3), we observed values similar to those detected in neurons from brains of neonatal rats [64] and mice [65].

The progressive increase of peak of inward Na^+^ currents over time also provided a valid parameter to confirm the maturation of a cell towards neuronal characteristics, becoming more negative with time. Moreover, consistent with expression of Na_v_, they were blocked by TTX which is known to selectively bind to NA_v_. Similar results have been obtained by other authors [31], though they observed the cells at earlier stages. On the contrary, outward K^+^ currents did not vary throughout development. This observation is consistent with results obtained on the development of K^+^ currents in human ESCs after neuronal differentiation reported by Belinsky et al. [27], although another group has reported a significant increase in K^+^ currents [31]. These discrepancies are probably due to differences in the time period analysed and method of differentiation. Finally, the characteristics of the APs in response to current injections evolved over time. They became shorter, reaching lower levels of threshold and higher amplitude.

The culture system used in our studies was based on a standard method that depends on chemical inhibition of TGFβ signaling to induce anterior neural fates [17]. We modified the original differentiation protocol by including defined components to reduce variability. These modifications allowed for efficient differentiation of forebrain neurons as indicated by the markers FOXG1, FORSE-1 and Tbr1. Similar to other studies [27] we observed a heterogeneous population of neurons at different developmental stages. Some of those cells were able to fire a single AP as soon as day 32 and others were still unable to respond to depolarizing currents at day 55. Possible explanations for this variability are remaining variables in standardized procedures for differentiation and stochastic variations of individual cells in the progression towards neuronal lineage. Nonetheless, an increasing number of neurons were capable of firing multiple APs over time, mimicking physiological neuronal development of various brain regions. This is consistent with previous studies showing that rat motor neurons fire single AP at E15 and repetitive APs after birth [66], and more recent studies on human ESCs and human iPSCs [27], [31] demonstrating the ability of iPSCs-derived neurons of repetitive firing at later stage of development.

The increase in the number of cells able to respond with multiple APs following a long depolarizing step is also consistent with the observation that, at a critical point in development, the density of Na_v_ provides enough Na^+^ conductance for the generation of multiple APs upon adequate stimuli [27]. The phenomenon of the rebound APs is a further indication of neuronal development, which is commonly observed in both mature [67], [68] and developing neurons [69]. Consistent with these studies, we observed rebound APs in 18% AP-competent neurons. Since some rebound APs persisted after the perfusion with TTX (23%), they are likely to be due to post-inhibitory rebound depolarization related to low-voltage activated Ca^2+^ channels, or the activation of inward rectifier channels [46].

Developing neuronal networks exhibit spontaneous release of neurotransmitters, unrelated to the AP generation. mEPSCs are known to be important for development [70], since they contribute to determining the structure of neuronal networks [71], [72]. Thus, the measurement of this quantal release of neurotransmitter is an indicator of the functionality of the neural network generated from human iPSCs. Consistent with this interpretation, the presence of mEPSCs *in vitro* in cells differentiated from iPSCs or directly from somatic cells has been recently reported [31], [73]. In these studies mEPSCs were found to occur sporadically and their evolution over time was not investigated. In our studies we first observed mEPSCs at day 32, with lower frequency and amplitude than commonly observed in neurons *in vitro* [74]. At this early stage of development mEPSCs were blocked by the reversible GABA_A_ antagonist bicucullin, suggesting that during early maturation of iPSC-derived neurons synaptic activity may rely on GABAergic neurotransmission. Interestingly, this finding is consistent with the observation that GABA is excitatory in the immature brain as a result of a high intracellular concentration of chloride (for a review see [75]). At t3, 21% of the cells had spontaneous events, and both amplitude and frequency increased over time. Moreover, consistent with the observation that glutamatergic synapses are formed after the GABAergic ones (for a review see [75]), synapses acquired glutamatergic properties at t3. These findings suggest that the synaptic apparatus is maturing over time in iPSC-derived neurons, recapitulating fundamental features of synaptic development.

Synaptic dysfunction plays a key role in neurodegenerative disorders such as Alzheimer’s [76], [77], Parkinson’s [78], [79], and Huntington’s [80]–[82] disease. Thus, the potential use of neurons differentiated from human iPSCs taken from patients to treat these diseases via cell replacement depends strongly on the ability of these cells to establish functional connections and mature synapses. A better understanding of the maturation of the synapses *in vitro* is also crucial for modelling the physiological conditions of maturation of neural progenitor cells transplanted *in vivo*. In addition, disease modelling of neurodegenerative disorders would best be performed in cells that accurately reflect *in vivo* physiology. It is therefore important to elucidate the mechanisms that promote the formation of neural networks and to record mEPSCs in these cells. Since cells plated on POL showed spontaneous events at a lower frequency, we tried to improve their maturation through co-plating with neonatal mouse glial cells. A previous study has shown accelerated spontaneous activity in neurons in maturation from stem cells via astrocyte co-culture [83]. We found that at the same day of neuronal differentiation *in* *vitro*, neurons plated with glia were more mature than those plated on POL. This was demonstrated by a more negative RMP and a higher percentage of cells responding with AP firing after a depolarizing current in co-cultures (Table 1). Moreover, at the same time point, more cells exhibited mEPSCs, and a 3-fold higher value of activity frequency was recorded. This may be in part due to increases in Ca^2+^ release by astrocytes. Astrocytic Ca^2+^ release in the extracellular space has been reported to trigger an increase in frequency of spontaneous excitatory postsynaptic AMPA currents in CA1 neurons, as well as activate extrasynaptic NMDA receptors [56], [84]. Another important role of glial cells is their release of important neurotrophic factors, such as Glial cell-Derived Neurotrophic Factor, that it is likely to facilitate improved neuronal maturation [85], [86]. Moreover, glial cells are important for glutamate uptake [87], [88]. Thus, these findings on co-cultures suggest that this method is useful to improve neuronal maturation and synaptic functionality.

In summary, our data demonstrate that the electrophysiological properties of human iPSC-derived neurons mature over time. The pathophysiological relevance of studies performed from neurons derived from stem cell technology should be properly evaluated by taking into account the electrophysiological status of the newly generated neurons. Most importantly, our findings help to establish baseline conditions for comparisons in future studies using neurons derived from stem cells both in basic and translational research of neurologic disorders. Additionally, the discovery of a failure or modification of an electrophysiological property of a neuron in a specific phase of maturation may be useful as a functional screen for the effects of known pathogenic mutations or a starting point for analysis of sporadic cases of disease with unknown genetic etiology. Thanks to the recent advances in lentiviral vector design for gene-modification of stem cells [89], [90], it may be possible to prevent or correct the misplaced expression of surface receptors, ion channels, or protein synthesis during the differentiation of human iPSCs toward mature neurons, in order to restore the physiological synaptic and excitatory/inhibitory responses of the cells.

## Supporting Information

Figure S1 Additional immunofluorescence markers. Neuronal cultures that had been differentiated for 54 days were immunostained as indicated. DNA is shown in blue. Images were taken with a 20X objective and the scale bar is 200 µm. A) A few scattered cells express the pluripotency marker Oct4. B) A minority of cells still express the neural progenitor marker nestin. C-D) Many cells express the forebrain marker FORSE-1 and the forebrain glutaminergic neuron marker Tbr1.
(TIFF)
