# Supplementary figures and images for: A Time Course Analysis of the Electrophysiological Properties of Neurons Differentiated from Human Induced Pluripotent Stem Cells (iPSCs)
Source: PLoS One. 2014 Jul 29;9(7):e103418. doi: 10.1371/journal.pone.0103418 (PMC4114788; doi:10.1371/journal.pone.0103418)

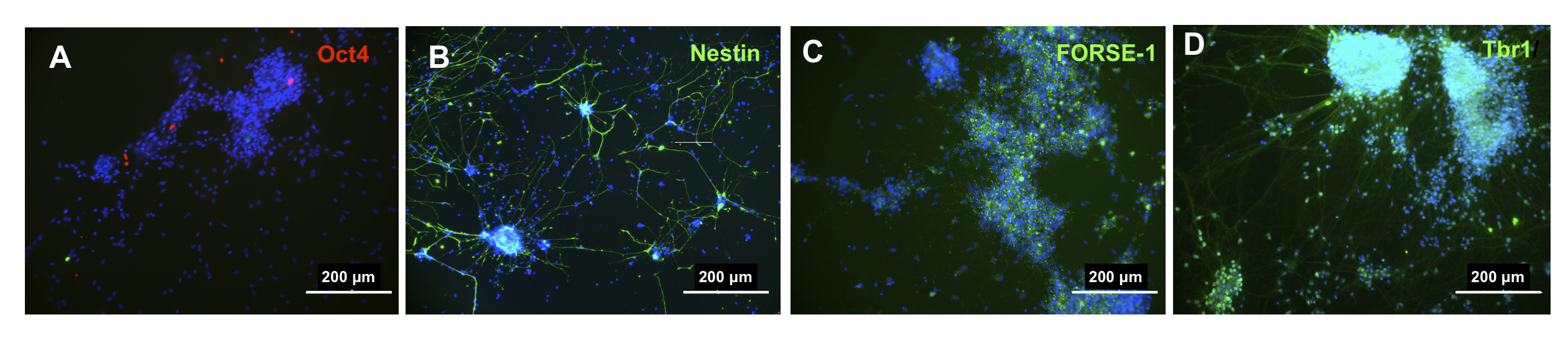

Supplement: Figure S1 — Additional immunofluorescence markers. Neuronal cultures that had been differentiated for 54 days were immunostained as indicated. DNA is shown in blue. Images were taken with a 20X objective and the scale bar is 200 µm. A) A few scattered cells express the pluripotency marker Oct4. B) A minority of cells still express the neural progenitor marker nestin. C-D) Many cells express the forebrain marker FORSE-1 and the forebrain glutaminergic neuron marker Tbr1. (TIFF) [file pone.0103418.s001.tiff]
